# Editorial: Early embryonic development: zygotic genome activation and epigenome reprogramming

**DOI:** 10.3389/fcell.2026.2003264

**Published:** 2026-10-16

**Authors:** Maki Asami, Bofeng Liu, Weikun Xia

**Affiliations:** 1 Sir William Dunn School of Pathology, University of Oxford, Oxford, United Kingdom; 2 MOE Key Laboratory of Bioinformatics, New Cornerstone Science Laboratory, School of Life Sciences, Center for Stem Cell Biology and Regenerative Medicine, Tsinghua University, Beijing, China; 3 Tsinghua-Peking Center for Life Sciences, Beijing, China; 4 Krantz Family Center for Cancer Research, Massachusetts General Hospital, Mass General Brigham Cancer Institute and Harvard Medical School, Charlestown, MA, United States

**Keywords:** early embryo development, embryonic genome activation, epigenetic reprogramming, totipotency, zygotic genome activation

Early embryogenesis transforms two terminally differentiated gametes into a totipotent zygote. This transition requires extensive epigenome reprogramming at multiple layers to generate a transcriptionally competent genome in embryos soon after fertilization (Du et al., 2022; Solberg et al., 2024; Xu et al., 2025). The distinct and asymmetric epigenetic states of the oocyte and sperm are largely reset, although some epigenetic marks can be transiently or stably inherited.

In mammals, DNA methylation is globally erased from both parental genomes through active and passive mechanisms, while it is preserved at imprinting control regions and selected repetitive elements (Lee et al., 2014; Smith and Meissner, 2013; Wu and Zhang, 2014). Genome-wide DNA methylation is subsequently reestablished after implantation. Histone modifications undergo more complex, mark-specific reprogramming: non-canonical histone patterns in oocytes can be partially inherited, whereas most sperm-associated histone features are rapidly reset after fertilization. The embryonic histone landscape is then re-established during pre- or post-implantation development, with active marks generally acquiring canonical patterns before repressive marks. Chromatin accessibility and 3D chromatin architecture are also extensively remodeled during this period, exhibiting a highly permissive and less structured state in the early stage. A key challenge is to determine how these regulatory layers are coordinated and how inherited versus newly established features influence developmental progression.

Early development initially relies on maternal RNAs and proteins deposited in the oocyte, with developmental control progressively shifting to the embryonic genome during the maternal-to-zygotic transition (Schulz and Harrison, 2019; Sha et al., 2020). Zygotic/embryonic genome activation (ZGA/EGA) represents the central event in this transition and occurs in successive waves whose timing, the genes activated, and the upstream transcriptional regulators differ substantially across species and are often poorly conserved (Ko, 2016). For example, the pioneer factor *Zelda* has long been recognized as a major activator of ZGA in *Drosophila* (Liang et al., 2008), whereas key mammalian ZGA/EGA regulators have been identified and functionally characterized only recently (Zou et al., 2022; Ji et al., 2023). These findings highlight the diversity of mechanisms that initiate ZGA across species, despite conserved physical constraints, such as the nucleocytoplasmic (N/C) ratio. How the early embryonic epigenome enables ZGA/EGA, and how nascent transcriptional activity subsequently reshapes the epigenome, remain poorly understood.

Technical advances have made these questions experimentally accessible. Low-input and single-cell transcriptomic and epigenomic technologies now resolve molecular changes in rare embryonic materials (Burton and Torres-Padilla, 2025; Kojima et al., 2025; Asami et al., 2023; Asami et al., 2022; Li et al., 2025). Sensitive translatomic, proteomic, and metabolomic approaches extend these analyses beyond RNA and chromatin (Cao et al., 2026; Zhu et al., 2025; Xiong et al., 2022). Genetic perturbation strategies and embryo-like models provide complementary systems for functional investigations (Ji et al., 2023; Mazid et al., 2022; Kagawa et al., 2022). The articles and reviews in this Research Topic use these conceptual and experimental advances to examine ZGA/EGA timing, epigenome remodeling, transposable-element regulation, metabolic control, and the orderly exit from totipotency.

Acker and Chen critically assess the nucleocytoplasmic-ratio model of ZGA/EGA timing (Acker and Chen). They integrate proposed mechanisms and cross-species evidence, concluding that the N/C ratio is one component of a broader regulatory network rather than a universal trigger. Ruiz reviews how transient totipotency-associated transcriptional programs are activated during ZGA and then silenced through coordinated transcriptional, epigenetic, and post-transcriptional mechanisms to enable the transition toward pluripotency (Ruiz). Wan et al. review epigenetic remodeling in mouse and human embryos, highlighting species-specific histone modification dynamics and their interplay with DNA methylation, chromatin accessibility, and three-dimensional genome organization (Wan et al.). Zheng et al. show that long-chain fatty acid beta-oxidation supports mouse preimplantation development by sustaining H3K18 acetylation and S-phase gene expression (Zheng et al.). Staubli et al. identify the maternal KRAB zinc-finger protein ZFPOBI1 as a regulator of RLTR10 endogenous retroviral elements in mouse oocytes and show that ERV structure and overlapping KRAB-ZFP targeting jointly constrain LTR-initiated transcription (Staubli et al.).

Together, these contributions highlight ZGA/EGA and epigenome reprogramming as interdependent rather than parallel processes. We thank all authors for their valuable contributions to this Research Topic and the editorial team for their time and expertise. We hope that this Research Topic will stimulate further studies linking multilayered regulatory mechanisms to molecular and developmental processes during the earliest stages of life.
